# Supplementary material for: Lytic/Lysogenic Transition as a Life-History Switch
Source: Virus Evol. 2024 Apr 3;10(1):veae028. doi: 10.1093/ve/veae028 (PMC11097211; doi:10.1093/ve/veae028)
Supplement: veae028_Supp [file veae028_supp.zip › LyticLysogenicSwitch-SupplementaryMaterial-try12aWithRefs.pdf]

Lytic/Lysogenic Transition as a Life-History Switch

Supplementary Material

Joan Roughgarden[1]

April 14, 2024

Terminology

A virus particle that infects a bacterium is a "bacteriophage" (eats bacteria) or just "phage", for short. A complete virus particle is a "virion"—it has a protein coat that is the "capsid" enclosing its nucleic acid genome.

A "virulent phage" enters a bacterial cell, commandeers its replication machinery and after a "latency period", releases copies of itself into the environment, killing the bacterium. "Lysis", from the Latin word for "a loosening", is the process by which the copies are released. The set of released copies is the "burst", the number of copies within the burst is the "burst size".

A "temperate phage" may either reproduce new virus particles for release into the environment while killing the bacterium *or* may integrate into the bacterium's genome and reproduce jointly with it. In the former case, the virus, like a virulent virus, commandeers the bacterium's replication machinery to produce copies of itself that after a latency period lyse from the bacterium killing it. This sequence is the "lytic pathway." In the later case, the virus integrates into the bacterial genome becoming a "prophage" that replicates along with the bacterial genome as the bacterium reproduces. The bacterium is not killed. A bacterium containing a prophage within it is a "lysogen". This sequence is the "lysogenic" pathway.

Following incorporation into a bacterium's genome, a temperate phage may either remain incorporated as a prophage during subsequent bacterial cell divisions. *Or*, it may become virulent by de-incorporating from the bacterium's genome, a process called "induction" and proceed to produce new virus particles by lysis into the environment while killing the bacterium it formerly resided in.

A prophage may augment a bacterium whose genome it resides in by providing the bacterium with a novel gene expressed through a process of "lysogen conversion" *i.e.*, the bacterium is converted from lacking a function into expressing a function. Prophages often encode genes called "morons" that are not directly involved in viral replication and can confer a benefit to their bacterial host. Such genes are independent transcriptional units of DNA that are expressed while the phage is in the prophage state. Morons can enhance the virulence of bacteria to their

[1]Hawaii Institute of Marine Biology, University of Hawaii, joaner@hawaii.edu and Department of Biology, Stanford University, rough@stanford.edu, ORCID: https://orcid.org/0000-0002-6360-7964.

own hosts by supplying phage-encoded toxins.

Terms in virology are defined relative to experimental protocols that have no ready counterpart in ecology, hindering the translation of terms between these disciplines. Virologists define the "multiplicity of infection" or MOI with a protocol whereby phage are added to bacteria in a medium. The ratio of the number of phage to the number of bacteria initially added to the medium is the MOI. In this paper, $v(t) = V(t)/N(t)$, is the ratio of virus particles, $V(t)$, to bacteria, $N(t)$, in their source pools at time, t. Hence, the MOI roughly corresponds to $v(0)$ in this paper.

A "one-step growth curve" is a curve of viral abundance in continuous time from the time of absorption until the time of lysis. The one-step curve may include information about the abundance of virus both within the bacteria as well as in the media. A "multi-step growth curve" is an experimental iteration of the one-step curve obtained by allowing the experiment to run beyond the first step. In this paper, viral abundance, $V(t)$, and bacterial abundance, $N(t)$, are curves in discrete time and are recorded solely at the end of each latency period, not within the latency period. They refer to abundance in the environmental source pools, not to virus within bacteria during a time step. Furthermore, Gadagkar and Gopinathan (1980) define an "effective multiplicity of infection" as the "mean number of phages adsorbed per infected bacterium". They also define the "effective burst size" as the "mean number of phages liberated per phage adsorbed." In this paper, at each time step, m indexes the number of virus in a bacterium where $b(m)$ is the burst size from a bacterium infected with m virus, and although not used here, $b(m)/m$, would represent the effective burst size.

Campbell Model of Virulent Phage/Bacteria Population Dynamics

In 1961, Allan Campbell proposed a mathematical model for the population dynamics of virulent phage and bacteria. The Campbell model (Campbell 1961) introduced features that have persisted in phage/bacteria models until the present day. The Campbell model is:

$$\frac{dN(t)}{dt} = r\,N(t)\left(1 - \frac{N(t)}{K}\right) - c\,V(t)\,N(t) \tag{S1}$$

$$\frac{dV(t)}{dt} = b\,c\,V(t-\tau)\,N(t-\tau) - c\,V(t)\,N(t) - d\,V(t) \tag{S2}$$

where $N(t)$ is the number of bacteria at time t, $V(t)$ is the number of virus particles at time t, $N(t-\tau)$ is the number of bacteria at time $t-\tau$, $V(t-\tau)$ is the number of virus particles at time $t-\tau$, τ is a time lag, r is the bacterial intrinsic rate of increase, K is the bacterial carrying capacity within its environment, c is the rate of conversion of mass-action collisions between virus particles and bacteria into infections, b is the burst size, and d is the per capita disintegration rate of virus particles. (The Campbell model was originally written for a phage/bacteria system in a chemostat. Here the chemostat nutrient flow terms are omitted.)

The Campbell model is a pair of nonlinear delay-differential equations. The first term of Eq. S1 assumes the bacteria grow logistically in the absence of virus. The second term is the loss of bacteria from infection brought about by mass-action random collision of bacteria with virus particles. The first term in Eq. S2 is the production of new virus particles that depend on the collisions that took place τ units of time previously. The time lag, τ, called the latency, is the time needed for the infecting virus particles to manufacture and release new virus particles using the bacteria's nucleotide replication machinery. The next term indicates the loss of free virus particles from the infections taking place at the present time, t. Thus, the virus particles disappear for the duration of the latency period. After infecting bacteria at time $t - \tau$ they reappear at time t as newly minted virus particles.

The Campbell model treats the bacteria and virus as *two* separate populations. This type of model is distinguished from those used in epidemiology that pertain to a *single* population containing multiple classes (Kermack and McKendrick 1927, Anderson and May 1979, Keeling and Rohani 2008, Diekmann *et al*. 2013, Bjørnstad 2018.) The time units in single-population models pertain to the time course of the disease in the host, say days to months, whereas the time units in the Campbell model relate to the kinetics of bacterial and viral replication, say minutes. Some models have been proposed for marine virus and also for bacteria in chemostats that are hybrids between the Campbell-type population model and an epidemiology type model (Beretta and Kuang 1998, 2001, Bull *et al*. 2006).

Equilibrium Coexistence of Virus and Bacteria

The equilibrium point of virus-bacteria coexistence in the Campbell model, $(\hat{N}, \hat{V})$, is found by setting $dN(t)/dt = 0$, $dV(t)/dt = 0$, $\hat{N} = N(t) = N(t-\tau)$, and $\hat{V} = V(t) = V(t-\tau)$ in Eqs. S1 and S2 yielding

$$\hat{N} = \frac{d}{(b-1)\,c} \tag{S3}$$

$$\hat{V} = \frac{r\big((b-1)\,c\,K - d\big)}{(b-1)\,c^2\,K} \tag{S4}$$

The equilibrium point is independent of the latency time, τ, although τ does influence the stability of the equilibrium. The condition for a feasible equilibrium point of coexistence between virus and microbe is read off from the numerator of Eq. S2 as

$$(b-1)\,c\,K > d \tag{S5}$$

This condition means the birth of virus has to exceed the death of virus. This condition can always be satisfied if K is large enough. Specifically, the bacterial carrying capacity, K, must exceed

$$K_{\min} = \frac{d}{(b-1)\,c} \tag{S6}$$

Table S1: Example Parameters in Campbell Model

r	0.0347
K	10^8
τ	15
b	40
c	10^{-4}
d	0.693

for the virus and bacterial populations to have positive equilibrium values. In fact, K_{min} works out to be the same as $\hat{N}$. Therefore, K must merely be greater than the bacterial abundance expected in the presence of the virus for the equilibrium in Eqs. S1 and S2 to be positive. The $\hat{N}$ is independent of r and K, indicating that its abundance is controlled by the virus' parameters. The $\hat{V}$ does depend on r and K, and increases with the bacteria's r and decreases with the bacteria's K. This dependence of the bacterial abundance on the properties of the virus and of virus abundance on properties of the bacteria is a well known property of Volterra-style predator-prey models in ecology (*c.f.* Roughgarden, 1998, p. 266 *ff*).

Parameters for Campbell Model

Order-of-magnitude guesses at parameter values for the model may be constructed from the literature. Consider the parameters for bacterial logistic growth, r and K. In the laboratory, the doubling time of *E. coli* is 20 minutes although it can be much slower in the wild (Gibson *et. al.* 2018). Assuming time units of minutes, this laboratory doubling time corresponds to $r = 0.0347$ (*i.e.* $0.0347^{20} = 2.00$). Next, in the human gut, which is the habitat of *E. coli*, this bacterium is typically present in 10^6 colony forming units per gram of stool (Foster-Nyarko and Pallen 2022). A typical value for the amount of faeces in a human is one lb or 450 grams (Alexa Answers, Amazon, retrieved July 21, 2022). So, a possible value for K is 10^8.

The time between the viral adsorption and first appearance of viral progeny is 10–15 minutes (Endy *et al.* 1997, Purohit *et al.* 2005). So, a possible value for the latency, τ, is 15 minutes.

The burst size from microbes infected with about two virus is about 80 virus particles (Gadagkar and Gopinathan 1980, Figure 4), so b will be taken as 40.

About one in 10^3–10^4 collisions between a virus and bacterium lead to a specific binding between the cellular receptor and the virus attachment protein on the surface of the virus (Philipson 1983). So in the model c is taken as 10^{-4}.

The half-life of free infectious influenza A virus is ≈ 3 hours (Baccam *et al.* 2006). If the half life is taken on the order of 1 hour, the disintegration rate of free virus, d, works out to be about 0.693 (*i.e.* $d = -\ln(1/2)$).

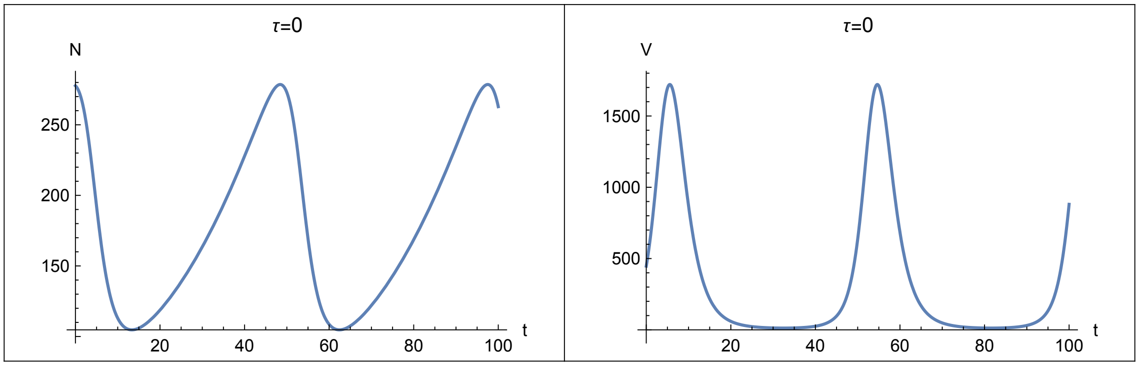

Figure S1: *Virus-Bacteria oscillation from the Campbell model with parameters from Table S1 excepting* $\tau = 0$. *Left is number of bacteria* vs. *time and right is number of virus* vs. *time. Initial conditions are* $N(0) = \hat{N} + 100$ *and* $V(0) = \hat{V} + 100$. *The period of the oscillation is about 55 minutes. Integration is from t = 0 to 100 minutes.*

These guesses at plausible parameter values are summarized in Table S1.

Guesses at plausible parameter values are summarized in Table S1. Using these parameters, the equilibrium from Eqs. S1 and S2 at which bacteria and virulent virus coexist out to be

$$\hat{N} = 177.692 \tag{S7}$$

$$\hat{V} = 346.999 \tag{S8}$$

Stability of Equilibrium with No Latency, $\tau = 0$

The coexistence of bacteria and a virulent virus require both that the equilibrium solutions for $\hat{n}$ and $\hat{v}$ from Eqs. S1 and S2 be positive *and* that the equilibrium point be stable to perturbations.

To begin, assume that the latency, τ, is 0, indicating an instantaneous conversion of virus infections into newly released virus particles. This assumption converts the Campbell model from a pair of delay-differential equations into a pair of Volterra-style ordinary differential equations that can be analyzed with conventional methods. The details of the analysis are recorded in the Supplementary Material.

With $\tau = 0$, the equilibrium is always stable. The trajectories reveal a virus-bacteria oscillation through time that winds in to the equilibrium point. The period of the oscillation is determined by d and r, independent of b, c and K. The oscillation speeds up as d and/or r are increased. A high K diminishes the stability of the virus-bacterial equilibrium point.

The Campbell model, Eqs. 1 and 2, with $\tau = 0$ can be integrated with the NDSolve[] function in *Mathematica*. The solutions appear in the time domain as a nearly endless virus-bacteria oscillation as illustrated in Figure S1 and in a parametric plot as a nested set of loops, each representing a different initial condition, as illustrated in Figure S2. If the bacterial carrying

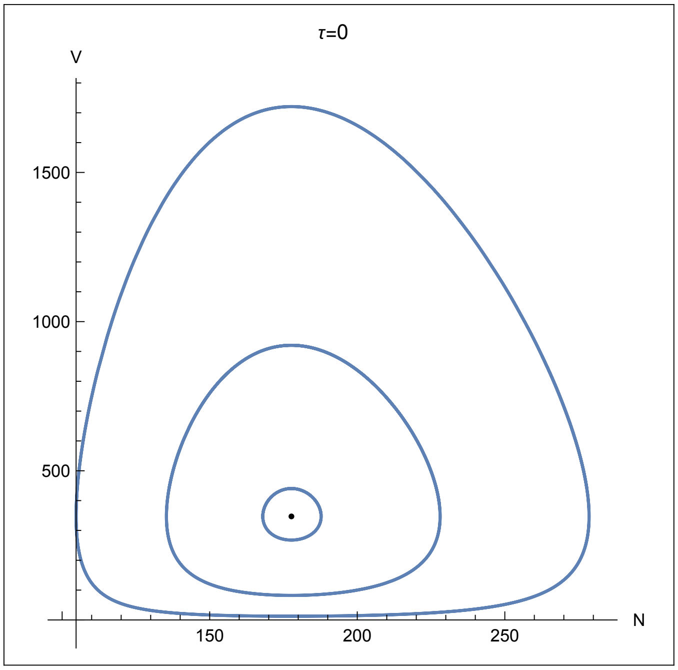

Figure S2: *Parametric plot of the Campbell model with parameters from Table S1 excepting* $\tau = 0$*. Horizontal axis is number of bacteria, vertical axis is number of virus, dot in center is the equilibrium point,* $(\hat{N}, \hat{V})$*. Outer loop was started at* $(N(0) = \hat{N} + 100,\ V(0) = \hat{V} + 100)$*, middle loop started at* $(N(0) = \hat{N} + 50,\ V(0) = \hat{V} + 50)$*, and inner loop started at* $(N(0) = \hat{N} + 10,\ V(0) = \hat{V} + 10)$*. Integration is from t = 0 to 100 minutes.*

capacity within the host is arbitrary set lower than 10^8, say approaching K_{min} from Eq. S4, then the equilibrium becomes increasingly stable and the trajectories more rapidly spiral into the equilibrium point (not illustrated).

Stability Analysis of Campbell Model with $\tau = 0$

The Jacobian matrix, J, with $\tau = 0$ evaluated at the equilibrium yields

$$J = \begin{pmatrix} \frac{-d\,r}{c\,K\,(b-1)} & -\frac{d}{b-1} \\ r\left(b - \frac{d}{c\,K} - 1\right) & 0 \end{pmatrix} \tag{S9}$$

The eigenvalues of J, λ_1 and λ_2 are a pair of conjugate complex numbers the real part of which is

$$\Re(\lambda) = -\frac{d\,r}{2\,(b-1)\,c\,K} \tag{S10}$$

Because $\Re(\lambda)$ is always negative, the equilibrium is always stable. And because the eigenvalues are complex, the trajectories reveal a virus-bacteria oscillation through time that winds in to the

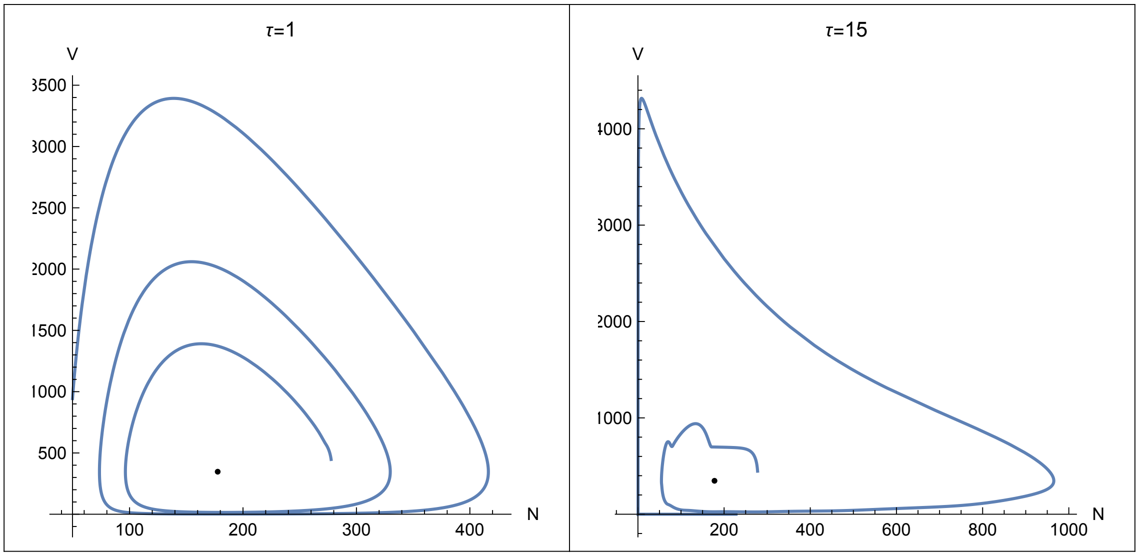

Figure S3: *Left: Parametric plot of the Campbell model with parameters from Table S1 excepting latency,* $\tau = 1$ *minute. Right: Parametric plot of the Campbell model with parameters from Table S1. Horizontal axis is number of bacteria, vertical axis is number of virus, dot in center is the equilibrium point,* $(\hat{N}, \hat{V})$*. Initial condition is* $(N(0) = \hat{N} + 100,\ V(0) = \hat{V} + 100)$*. Integration is from t = 0 to 150 minutes on the left and to 400 minutes on the right.*

equilibrium point in a flow pattern representing a "stable focus". The period of the oscillation is approximately

$$T = \frac{e\,\pi}{\sqrt{d\,r}} \tag{S11}$$

The period is determined by d and r, independent of b, c and K. The oscillation speeds up as d and/or r are increased.

Even though $\Re(\lambda)$ is always negative, its magnitude varies inversely with the bacterial carrying capacity, K. Hence, a high K diminishes the stability of the virus-bacterial equilibrium point. Indeed, if K is infinite, the equilibrium point is neutrally stable and the flow of trajectories represents a "center" as occurs in the original Volterra predator-prey model (Volterra 1926; for a summary *cf*. https://en.wikipedia.org/wiki/Lotka–Volterra_equations, retrieved July 28, 2022).

With the parameters of Table S1,

$$\Re(\lambda) = -3.08 \times 10^{-8} \tag{S12}$$

$$T = 55.07 \text{ minutes} \tag{S13}$$

The $\Re(\lambda)$ for these eigenvalues is nearly zero because k is 10^8 in Table S1 and thus the equilibrium is, practically speaking, neutrally stable.

Stability of Equilibrium with Latency, $\tau > 0$.

The introduction of the latency time lag, τ, destabilizes the equilibrium at which virus and bacteria coexist. Although stability conditions for a system of delay-differential equations cannot generally be analyzed mathematically, the NDSolve[] function in *Mathematica* can successfully numerically integrate the Campbell model with a fixed time lag, τ. The left side of Figure S3 shows the parametric plot of virus and bacteria through 150 minutes with a small latency, $\tau = 1$ minute. The virus and bacteria populations were started toward the right of the equilibrium point, at $N(0) = \hat{N} + 100$ and $V(0) = \hat{V} + 100$, respectively. The trajectories wind out from the initial condition, spiraling ever farther from the equilibrium point.

The dynamic instability caused by the latency period becomes even worse with the realistic latency period of $\tau = 15$ minutes, as was recorded in Table S1. The right side of Figure S3 shows the parametric plot of virus and bacteria through 400 minutes for a 15 minute latency. The virus and bacteria populations were again started toward the right of the equilibrium point, at $N(0) = \hat{N} + 100$ and $V(0) = \hat{V} + 100$, respectively. The trajectories show a jagged start because the initial condition for a delay-differential equation should specify the value of the variables at time $t = 0$ *and* at all times in the past until $t = -\tau$. Because the value of the variable is unknown before $t = 0$, the value at $t = 0$ is attributed to the earlier times. After a while, the trajectory catches up with its history and the trajectory smooths out. Thereafter the trajectories wind out and quickly hug the axes.

Campbell in 1961 may not have been aware of the destabilizing role of the viral latency period. At that time he wrote "If a virulent phage and a susceptible bacterium are mixed in an open growth system, such as a chemostat, one expects that, in general, the concentration of the two will approach some stable steady state value." (Campbell 1961, p157–8). But twenty years later, in 1981, he wrote that "Stable coexistence of phage and bacteria in chemostats has been verified experimentally. Comparison of theory with experiment revealed one discrepancy: Although the concentrations of bacteria and phage stabilized at approximately the predicted values, theoretical analysis and computer simulations predicted unstable oscillation rather than a stable steady state." (Campbell 1981, p. 61). Indeed, researchers testing a version of the Campbell model with chemostats in 1977 wrote, "the time lag due to the latent period of the phage has a destabilizing effect which may be stronger than the opposite, stabilizing, effect of [bacterial population] regulation (Levin *et. al*. 1977, p. 11).

Critique of Campbell Model

Fifty-six years after the ground-breaking Campbell (1961) model appeared, Krysiak-Baltyn *et al.* (2017) published an extensive review of models for virulent phage and bacteria. They observed that "The basic model initially suggested by Campbell, and later refined by Levin and colleagues

[Levin *et al*. 1977], has remained in use since first publication. Later studies have mostly made minor adjustments to the existing model to study dynamic behavior." (p. 964) They further observed that "Earlier studies on simple chemostat models indicated that the dynamics of the model are not in good quantitative agreement with experimental data. In particular, the stability of the simulated models is lower than expected, with many exhibiting heavy oscillations and extinction events where stable steady-states exist experimentally." (p. 964) Krysiak-Baltyn *et al.* (2017) conclude that "On a practical level, the usefulness of computational models of bacteria and phages has not yet been established. The simpler chemostat models may first need to become more accurate" (p. 965).

Thus, the Campbell (1961) model suffers quantitative limitations. First, it predicts instabilities to virulent virus/bacteria population dynamics that are contradicted experimentally. Second, the Campbell (1961) is so mathematically challenging that its use is impractical in biology. How to mathematically analyze a system of nonlinear delay-differential equations is an ongoing subject of research in applied mathematics (Tang and Zou 2008, Huang *et al.* 2010; indeed many investigators simply put the time lag, τ, equal to zero to evade the problem, converting the delay differential equations into ordinary differential equations). And as discussed in the text of the main document, the qualitative picture provided by the Campbell model is problematic. The Campbell model envisions that virus/bacteria population dynamics involves two counteracting forces—the stabilizing force of density-dependent bacterial population growth and the destabilizing force of time lags arising from the virus latency period. The outcome is a balance of these forces. This qualitative picture might not be accurate.

Density-Dependent Burst Size

Several choices are available for the burst size from a bacterium as a function of the number of virus in it, $b(m)$. The burst size might increase linearly with m so that $b(m) = b_o\, m$ where b_o is the contribution to the overall burst made by a single viral particle (Patel and Rao 1984, Figures 1–2). This case is for no density dependence. Alternatively, $b(m)$ might be a constant, $b(m) = b_o$, indicating that the burst size does not increase for m beyond the initial infection. A constant $b(m)$ indicates strong density dependence and is consistent with surveys reporting that burst sizes are characteristic of various viral taxa (Parada *et al.* 2006, Ranasinghe 2019). Another possibility is to assume $b(m)$ is a convex increasing function of m (Gadagkar and Gopinathan 1980, Figures 4–5). In this case, the burst-size function might be taken as $b(m) = b_o\, m^\alpha$ where α is an exponent between 0 and 1, say 1/2. A final possibility is to allow α to be negative, say $\alpha = -1$, to indicate that burst size varies inversely with the multiplicity of infection, indicating extremely strong density dependence as a response to "superinfection" (Brown and Bidle 2014). All these possible relations between burst size and m may be subsumed by taking $b(m) = b_0\, m^\alpha$

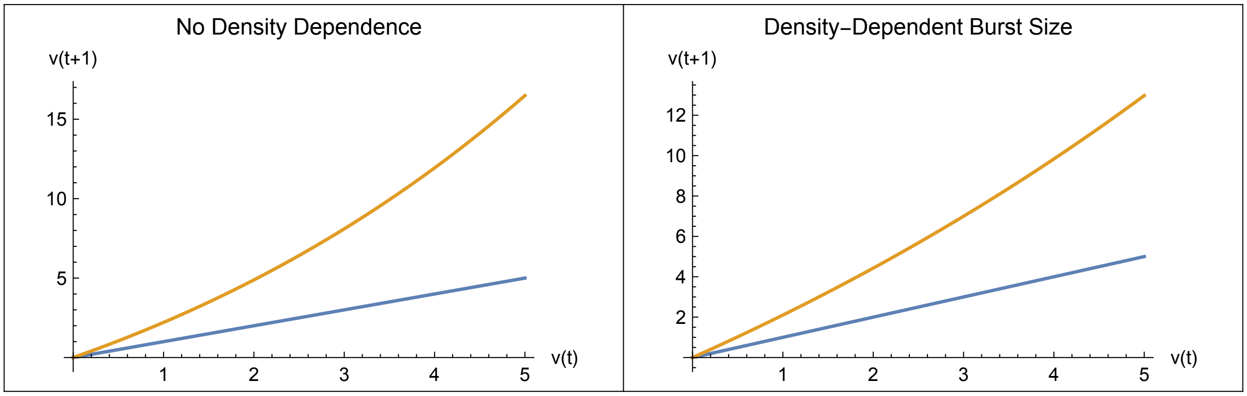

Figure S4: *$v(t+1)$ vs. $v(t)$, in brown, for no density dependence (left) and for a density-dependent burst size (right) from Eqs. 10 and 13, respectively. The diagonal line, in blue, indicates $v(t+1) = v(t)$. An intersection of the brown curve with the blue line would indicate an equilibrium point where virus and bacteria coexist, provided the equilibrium is stable. Because the curve does not intersect the line in either plot, no coexistence occurs in either case. Here, $a_o = 0.1$, $b_o = 40$, and $R = 2$*

with α as 1, 1/2, 0, or -1. Here, the cases of $\alpha = 1$ and $\alpha = 0$ will be investigated analytically. Results (not shown) for other α are similar.

No Density Dependence: $a(v(t)) = a_o$**,** $b(m) = b_o\, m$

Here,

$$\mu(t) = a_o\, v(t) \tag{S14}$$

and, upon doing the summation in Eq. 4 ,

$$\widetilde{b}(\mu(t)) = b_o\, a_o\, v(t) \tag{S15}$$

so Eq. 5 becomes

$$v(t+1) = \frac{b_o}{R}\, \frac{a_o\, v(t)}{e^{-a_o\, v(t)}} \tag{S16}$$

Analysis of this case appears in Figure S4 (Left).

Density-Dependent Burst Size: $a(v(t)) = a_o$**,** $b(m) = b_o$

Here,

$$\mu(t) = a_o\, v(t) \tag{S17}$$

and, upon doing the summation in Eq. 4,

$$\widetilde{b}(\mu(t)) = b_o\, (1 - e^{-a_o\, v(t)}) \tag{S18}$$

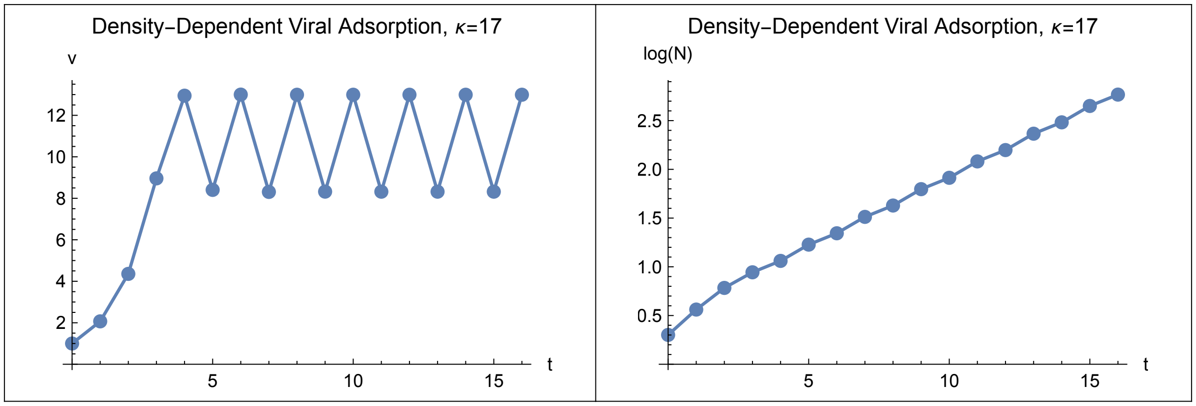

Figure S5: *Left: Trajectory of $v(t)$ showing a two-point limit cycle around the unstable equilibrium at $\hat{v}$. Right: Trajectory of* $\log_{10}(N(t))$ *showing a two-point oscillation in the exponential bacterial population growth. Here,* $\kappa = 17$, $v(0) = 1$, $N(0) = 2$, $t_{\max} = 16$, $\kappa = 12$, $a_o = 0.1$, $b_o = 40$ $R = 2$ *and* $t_{\max} = 16$.

so Eq. 5 becomes

$$v(t+1) = \frac{b_o}{R} \frac{1 - e^{-a_o v(t)}}{e^{-a_o v(t)}} \tag{S19}$$

Analysis of this case appears in Figure S4 (Right).

A point of possible coexistence occurs at the value of v for which $v(t+1)$ equals $v(t)$. This condition is shown graphically in Figure S4. An intersection of the curve for $v(t+1)$ with the line for $v(t+1) = v(t)$ would indicate an equilibrium point for possible virus/bacteria coexistence. The curve and line do not intersect in either case. Therefore coexistence, not surprisingly, is impossible without any density dependence, but surprisingly, is also impossible if the density dependence is solely in the burst size assuming the function relating burst size to density is $b(m) = b_0\, m^{\alpha}$. (Illustrated only for $\alpha = 0, 1$.)

Trajectories with $\kappa > 14$

Figure S5 illustrates trajectories of both virus and bacteria for $\kappa > 14$. Trajectories illustrate a two-point limit cycle of $v(t)$ around the unstable $\hat{v}$. The trajectory of $N(t)$ also shows a two-point oscillation around the asymptotic growth factor although again, the scale on the graph makes the oscillations difficult to see.

The existence of a two-point limit cycle around the unstable equilibrium in Figure S5 is consistent with known dynamics for the discrete-time logistic equation (May 1976.) The possibility of more complex oscillations and chaotic trajectories with $\lambda_+ < -1$ remains to be explored. For the purposes of this paper, a comparison of the oscillations for this model in Figure S5 with those in Figures S1–S3 shows that the oscillations in the present model are bounded from above and below whereas those in the Campbell (1961) model approach arbitrarily close to the axes,

implying population extinction.

Poisson Summation Identities

$$\sum_{m=1}^{\infty} P_m(\mu) = 1 - e^{-\mu} \tag{S20}$$

$$\sum_{m=1}^{\infty} m\, P_m(\mu) = \mu \tag{S21}$$

References

Anderson, R. M. and May, R. M. 1979. "Population Biology of Infectious Diseases: Part I." Nature 280, 361–367.

Campbell, Allan. 1961. Conditions for the Existence of Bacteriophage. *Evolution* 15:153–165.

Campbell, Allan. 1981 Evolutionary Significance of Accessory DNA Elements in Bacteria, Ann. Rev. Microbiol. 35:55–83

Baccam P, Beauchemin C, Macken CA, Hayden FG, Perelson AS. 2006. Kinetics of Influenza A Virus Infection in Humans. J Virol 80:7590–7599, https://doi.org/10.1128/jvi.01623-05

Beretta, E. and Y. Kuang 1998. Modeling and analysis of a marine bacteriophage infection. Mathematical Biosciences 149 57–76

Beretta E. and Y. Kuang. 2001. Modeling and analysis of a marine bacteriophage infection with latency period. Nonlinear Anal Real World Appl 2:35–74.

Bjørnstad, Ottar 2018. Epidemics: Models and Data using R. Springer Nature Switzerland AG. https://doi.org/10.1007/978-3-319-97487-3, Second Edition, 2023.

Brown, Christopher M. and Kay D. Bidle. 2014. Attenuation of virus production at high multiplicities of infection in *Aureococcus anophagefferens*. Virology 466-467 (2014) 71–81. http://dx.doi.org/10.1016/j.virol.2014.07.023

Bull, J. J., J. Millstein, J. Orcutt and H. A. Wichman 2006. Evolutionary Feedback Mediated through Population Density, Illustrated with Viruses in Chemostats. The American Naturalist , Vol. 167, No. 2 pp. E39–E51 https://www.jstor.org/stable/10.1086/499374

Diekmann, Odo, Hans Heesterbeek and Tom Britton 2013. Mathematical Tools for Understanding Infectious Disease Dynamics. Princeton University Press. Volume 7 in the series Princeton Series in Theoretical and Computational Biology
https://doi.org/10.1515/9781400845620

Endy D, Kong D, Yin J. 1997 Intracellular kinetics of a growing virus: a genetically structured simulation for bacteriophage T7. Biotechnol Bioeng. Jul 20 55(2):375–89.

Foster-Nyarko, Ebenezer and Mark J. Pallen 2022 The microbial ecology of *Escherichia coli* in the vertebrate gut. FEMS Microbiology Reviews, 46, 1–22 DOI: 10.1093/femsre/fuac008

Gadagkar, Raghavendra and K. P. Gopinathan 1980 Bacteriophage burst size during multiple infections. J. Biosci., Vol. 2, Number 3, September 1980, pp. 253–259.

Gibson B, Wilson DJ, Feil E, Eyre-Walker A. 2018 The distribution of bacterial doubling times in the wild. Proc. R. Soc. B 285: 20180789. http://dx.doi.org/10.1098/rspb.2018.0789

Huang, Gang, Yasuhiro Takeuchi, and Wanbiao MA. 2010. Lyapunov Functionals for Delay Differential Equations. Model of Viral Infections. Siam J. Appl. Math Vol. 70, No. 7, pp. 2693–2708

Keeling, M. J., & Rohani, P. (2008). Modeling infectious diseases in humans and animals. Princeton, NJ: Princeton University Press.

Kermack, W. O. and McKendrick, A. G. 1927 ”A Contribution to the Mathematical Theory of Epidemics.” Proc. Roy. Soc. Lond. A 115, 700–721.

Krysiak-Baltyn, Konrad , Gregory J. O. Martin, Anthony D. Stickland, Peter J. Scales, and Sally L. Gras 2016. Computational models of populations of bacteria and lytic phage. Crit Rev Microbiol, 2016; 42(6): 942–968 DOI: 10.3109/1040841X.2015.1114466

Levin, Bruce R. , Frank M. Stewart, and Lin Chao 1977 Resource-limited growth, Competition, and Predation: A model and Experimental Studies with Bacteria and Bacteriophage. Amer. Natur. 111:3–24.

May, Robert. 1976. Simple mathematical models with very complicated dynamics. Nature 261:459–467.

Parada, Verònica , Gerhard J. Herndl and Markus G. Weinbauer. 2006. Viral burst size of heterotrophic prokaryotes in aquatic systems. J. Mar. Biol. Ass. U.K. 86, 613–621

Patel, I. R. and K. K. Rao. 1984. Bacteriophage burst size as a function of multiplicity of infection. Current Science 53:198–200.

Purohit, Prashant K., Mandar M. Inamdar, Paul D. Grayson, Todd M. Squires, Janè Kondev, and Rob Phillips 2005 Forces during bacteriophage DNA packaging and ejection. Biophys J. Feb88(2):851–66. (p.852 caption to figure 1)

Ranasinghe, Nilakshi 2019. Literature study of virus size, burst size, latent period and genome size across different lytic eukaryotic and prokaryotic virus groups—an overview of traits and possible trade-offs. MSc Thesis. Department of biology, University of Bergen

Roughgarden, J. 1998. *Primer of Ecological Theory*. Prentice Hall. New Jersey.

Tang, X. H. and Xingfu Zou. 2008. Global Attractivity in a Predator-Prey System with Pure Delays. Proceedings of the Edinburgh Mathematical Society 51: 495–508 DOI:10.1017/S0013091506000988

Volterra, V. (1926). Variazioni e fluttuazioni del numero d'individui in specie animali conviventi. Mem. Acad. Lincei Roma. 2: 31–113.
